# Science of quality improvement – from vision to reality: Experience from a leading academic healthcare center in Qatar

**DOI:** 10.5339/qmj.2022.18

**Published:** 2022-03-12

**Authors:** Muhammad Zahid, Adeel Ahmad Khan, Zohaib Yousaf, Ahmed Ali A A Al-Mohammed, Dabia Hamad S H Al Mohanadi

**Affiliations:** ^1^Department of Internal Medicine, Hamad Medical Corporation, Doha, Qatar E-mail: adeel_1026@yahoo.com; ^2^Department of Endocrinology, Hamad Medical Corporation, Doha, Qatar

**Keywords:** quality improvement curriculum, patient safety, healthcare center, Qatar

## Abstract

Quality improvement and patient safety are cornerstones to the delivery of effective patient care. The introduction of a quality improvement curriculum for medical students and trainee physicians can have a significant effect on their understanding of the science of improvement and its role in improving the quality of healthcare delivery and patient safety. The article describes the development and implementation of a quality improvement curriculum for trainee physicians in the department of internal medicine at a tertiary care center in Qatar through online training courses, workshops, didactic activities, and trainee-led quality improvement projects.

## Introduction

The complexity of healthcare services stems from the inherent drive to improve and innovate. The improvement in a healthcare system is geared toward delivering a consistently safe, efficient, and timely care. Despite the efforts to deliver patient-centered care, patient safety remains a significant challenge. Avoidable errors are among the 10 leading causes of death and disability globally. Of the reported adverse events in a healthcare system, half of the events are avoidable.^1^ These numbers may underestimate the real incidence because of under-reporting. The Institute of Medicine, in its report titled “To Err Is Human: Building a Safer Health System,” provided a basic framework on how healthcare providers could reduce medical errors and provide safe, effective, and efficient care.^2^ Healthcare providers should understand the science of improvement to optimize outcomes of each care episode and overall outcomes in a healthcare system.

The old adage “a chain is no stronger than its weakest link,” applies to the modern healthcare systems. The science of quality improvement (QI) is a part and parcel of any modern training program.^3^ The Accreditation Council for Graduate Medical Education (ACGME) is the leading body setting institutional educational standards in the United States through its international arm (ACGME-I) globally. Practice-based learning and improvement is one of the seven core competencies of ACGME. Up to 72% of residents feel that the QI curriculum should be part of their training.^3,4^ The introduction of a QI curriculum can significantly improve medical students’ and residents’ knowledge about the quality of healthcare and patient safety.^5^ Therefore, graduate medical education and training programs that produce future leaders in healthcare must develop structured training in the science of improvement.

Formal and summative assessments during training ensure the progression of medical trainees. These assessments are mainly designed to assess theoretical and clinical knowledge. There is not enough emphasis on learning the science of improvement. In the United States, training programs have developed the quality improvement and patient safety (QIPS) curriculum over the last two decades, and QI training is now mandatory for medical students.^4^ In the United Kingdom, there is a renewed focus on audit, QI, and patient safety.^4^ QI and patient safety are the core competencies for healthcare providers as mandated by regulatory authorities such as the ACGME of the United States and the General Medical Council of the United Kingdom.^4^

### Types of QI curriculum

Globally, health educators and healthcare organizations need to establish learner-centered curricula to train healthcare professionals, with the ultimate goal of positively influencing the culture of QI and patient safety. Developing strong leadership and engagement of educators and staff is essential to understand the raison d'être and the change process. QI and patient safety should be an essential part of all training programs at the undergraduate and postgraduate levels. Medical schools in the United States and the United Kingdom have started introducing patient safety training into the undergraduate curriculum. This has shown improvement in knowledge, attitudes, and approach to patient safety.^6,7^

The World Health Organization (WHO), through its patient safety program, identified 11 key topics for QIPS curricula^8^ (Table 1). An increasing number of curricula are developed based on these topics.^9,10^ The QI curricula vary in the methods of disbursement, but it can be divided into the following three types: 1) educational sessions in didactic format with either a single prolonged session or multiple small encounters, 2) online teaching methodology primarily through structured online modules, and 3) combination of teaching activities and experiential learning whereby trainees participate in the administration of QI projects.^11^

All three methods have shown varied success in QI education. Moskowitz et al. reported a positive change in students’ knowledge and beliefs toward patient quality and safety after a 1-day QI training program.^12^ The use of web-based educational modules on patient safety for medical trainees led to an improvement in learning outcomes based on pre- and post-module test scores.^13^ Oyler et al. reported a significant improvement in residents’ QI skills after using a learning module, followed by implementation through practical QI projects.^14^ A combination of teaching and hands-on training programs has shown better outcomes than other methodologies.^15^

### Internal Medicine residency program (IMRP)

The Hamad Medical Corporation (HMC) is a tertiary care hospital and the primary healthcare provider in Qatar. It has a capacity of over 2000 beds and is accredited by the Joint Commission International. The IMRP at HMC is an ACGME-International (I) accredited program. IMRP has more than 200 residents and includes a combination of locally trained and foreign medical graduates. Teaching QI methodologies to trainees is a requirement for ACGME-I accredited programs.^4^ Structured training in the science of improvement allows trainees to understand systems, processes, and outcomes. This understanding helps in the identification of root causes and suggests changes to improve the system by applying QI concepts and skills.

### QIPS curriculum

A search performed on the PubMed using Boolean operator strategy of “Quality improvement” OR “QIPS” AND “Curriculum” AND (GCC OR “Middle East” OR MENA OR Bahrain OR Kuwait OR UAE OR Qatar OR “Saudi Arabia” OR Oman)” yielded 13 articles. The relevant articles focused on ethics, communication skills, story based-learning quality initiative, and a module-based QI initiative. No published article reported on a comprehensive well-established QIPS curriculum in any training program in the Gulf Cooperation Council area. The IMRP at HMC has developed the QIPS curriculum inclusive of didactics, workshops, and practical methods to demonstrate core knowledge of improvement science. These are delivered by physicians experienced and trained in QI. This system is designed for the unique needs of the local healthcare and training system and includes the following QI initiatives:

#### QIPS induction for new residents

QI and patient safety orientation are conducted by the IMRP for all new residents at the start of their training. Residents are oriented and participate in ongoing QI projects. On average, each QI project involves five residents. Each resident is assigned specific tasks in the conduct of the project. During their training, residents are part of committees tasked to implement QI initiatives such as risk management committees and clinical pathway committees.

#### Institute for healthcare improvement (IHI) open-school online courses

Understanding of QI is a requirement for progression through the residency training program. One of the objective measures for a trainee's understanding of QI is the successful completion of the IHI open-school QI courses. All residents are given the opportunity by the training program to develop their QI skills by receiving free-of-cost access to the IHI open-school online courses (six modules; Table 2).^16^ All IHI courses are completed within 2 years from the start of the residency program.

#### QIPS workshop

A 2-day QIPS workshop is conducted biannually to provide empirical learning opportunities for the trainees. This workshop educates the trainees on the following aspects:

 A. QI science basics to provide residents with basic knowledge to run small QI projects (microsystem projects).

 B. Build awareness of the underlying theories of QI, including the theory of profound knowledge, Juran theory, and reliability science

 B. Understand the difference between an audit and QI.

 D. Describe the concept of safety and harm in the healthcare context

 E.  Understand and describe the link between structure, process, and outcome

 F.  Understand the model for improvement and the plan–do–study–act cycle

 G. Develop and interpret run charts

 H. Understand the basics of advanced measurement charts, such as the control chart

 I.   Exercises like building an airplane factory to apply the model for improvement in a non-healthcare context practically

 J.  Understand transparency, culture of safety, and error reporting

A 2-day QI workshop was conducted in October 2020 for residents belonging to the IMRP at HMC, Qatar. An anonymous paper-based survey consisting of six questions designed on a 3-point Likert scale was distributed pre-workshop. The survey had two columns for answers marked as pre- and post-workshop. The participants were instructed to fill in the first column before the workshop. The second column was filled by the participants after the workshop. Of the 80 participants, 76 completed both columns of the questionnaire, leading to a response rate of 95%. Specifically, 52 participants were year 1, 18 were year 2, and 6 were year 3 trainees. Numbers between 1 and 3 were assigned to each option of every question in ascending order of the ordinal data; 1 was assigned to a negative answer, 3 to a positive answer, and 2 to an uncertain response. The measurement of central tendencies showed an increase in the mean score of the post-workshop survey across all categories compared with that of the pre-workshop survey. The most significant improvement was seen in the confidence of conducting a QI project, followed by knowledge of QI methods and familiarity with the QI resources and mentors’ availability. A paired t-test was performed for each question. The pairs were generated for each candidate based on each question's pre- and post-workshop scores. The criterion for the significance of a two-tailed t-test was a p-value of < 0.05. A significant difference between the pre- and post-workshop groups was noted across all six questions (Table 3).

#### Didactics and microlearning

Regular didactic sessions are arranged to cover the entire curriculum annually. Each 1-h long educational session is covered by the protected time for education as mandated by the Department of Graduate Medical Education. Patient care is fully taken over by the attending physicians, so the residents can focus on these crucial educational sessions. These short training sessions serve as microlearning opportunities, which break down complex concepts of the science of improvement with added benefit of repetition. Microlearning is a relatively newer educational strategy but is gaining popularity and is shown to be effective in healthcare education.^17^

#### QI morning report

A 1-h session focusing on the science of QI is also conducted once a month. The sessions are part of a series of lectures on various QI-related topics. The sessions also focus on the discussion of the ongoing resident-led QI projects with the rest of the trainees for mutual learning.

#### Error reporting system

An online error reporting system is available to all physicians and trainees throughout the institution. They are trained on how to report an error, with the option of reporting anonymously and non-anonymously. Residents in the internal medicine department are encouraged to report an error for the improvement of patient safety and quality of healthcare delivery.

#### Clinical care improvement training program (CCITP)

CCITP is a 1-year program designed by the Hamad Healthcare Quality Institute (HHQI, a part of HMC) to train healthcare professionals from healthcare institutions across Qatar in QI. This program imparts advanced knowledge critical in planning QI projects. This training is offered to trainees who show interest in QI and are expected to be future leaders in QI. It provides not only advanced tools and resources to the participants but also mentorship from the current leaders in QI along with direct interaction with policymakers, administrators, and stakeholders in QI. This workshop has resulted in multiple corporate-level projects.^18–20^

Some QI projects completed by residents at our institution are mentioned in Table 4. The success of the QI curriculum is assessed by evaluating the proportion of residents who have completed IHI online courses, attended the 2-day QI workshop, and participated in QI projects during the first 2 years of residency. In the batch of trainees starting in 2019, all residents have completed the IHI training modules, while more than 90% of the residents have attended the QI workshop and participated in QI projects within 2 years.

### Barriers to implementation of QI curricula

One of the global challenges of introducing a QI curriculum in the training programs is the resulting increase in workload on already overwhelmed trainees.^21^ Furthermore, the lack of clarity and methodology of the QI curriculum in implementing QI projects also poses a barrier to trainees’ participation in QI projects.^22^

These are the challenges of incorporating a QI curriculum into a residency training program:

• Time constraints at the level of faculty and residents’ interface
• Engagement of residents and faculty in QI work
• Training of residents and faculty in the science of improvement
• Changing the existing culture within the healthcare system
• Frustration associated with not achieving sustainable change
• Resource allocation for data collection
• Lack of funding

## Conclusion

The revamping of healthcare systems to deliver high-quality, safe care is imperative worldwide. Resources should not be wasted because of preventable errors and harms. Currently, QI and patient safety are essential core competencies for health professionals. Major reforms are required to translate patient safety science into daily practice. The strong commitment of leaders and healthcare educators is necessary to overcome the barriers and challenges of integrating patient safety into education and training.

The introduction of a QI curriculum for trainees at our institution based on QI workshops, online learning modules, and didactic activities has increased knowledge among medical residents about the science of improvement. This has led to the initiation of several QI projects led by trainee physicians, thereby contributing to the high standards of care and patient safety. Further innovations are still needed to implement the QIPS curriculum and translate it into daily clinical practice.

### Conflict of interest

Authors declare that there is no conflict of interest.

### Acknowledgments

None

### Funding statement

No funding was acquired for this manuscript

## Figures and Tables

**Table 1:** WHO QI curriculum guide

WHO curriculum guide topics

1. What is patient safety?

2. What are human factors and why they are important to patient safety?

3. Understanding systems and the effect of complexity on patient care

4. Being an effective team player

5. Understanding and learning from errors

6. Understanding and managing clinical risk

7. Introducing quality improvement methods

8. Engaging with patients and carers

9. Minimizing infection through improved infection control.

10. Patient safety and invasive procedures.

11. Improving medication safety


QI, quality improvement; WHO, World Health Organization

**Table 2:** IHI open-school online courses undertaken by the participants

Improvement capability	

QI 101	Introduction to healthcare improvement

QI 102	How to improve with the model for improvement

QI 103	Testing and measuring changes with the PDSA cycle

QI 104	Interpreting data: run charts, control charts, and other measurement tools

QI 105	Leading quality improvement

QI 201	Planning for spread: from local improvements to system-wide change


IHI, Institute for Healthcare Improvement; PDSA, plan–do–study–act

**Table 3:** Pre- and post-workshop survey (n = 76)

	Survey questions	Mean pre-workshop	Mean post-workshop	Mean difference	P-value

1.	How would you rate your knowledge of quality improvement methods? No knowledge = 1 Somewhat knowledgeable = 2 Very knowledgeable = 3	1.86 ± 0.354	2.34 ± 0.505	0.487	< 0.001

2.	How interested are you in being a part of a quality improvement project? Not interested = 1 Somewhat interested = 2 Very interested = 3	2.51 ± 0.529	2.63 ± 0.512	0.118	0.049

3.	How familiar are you with the quality improvement resources and mentors available at the institution? Not familiar at all = 1 Somewhat familiar = 2 Very familiar = 3	1.83 ± 0.473	2.26 ± 0.574	0.434	< 0.001

4.	Do you think a quality improvement curriculum is an integral part of a residency training program? No = 1 Unsure = 2 Yes = 3	2.76 ± 0.563	2.91 ± 0.372	0.145	0.002

5.	How confident are you to conduct a quality improvement project? Not confident at all = 1 Somewhat confident = 2 Very confident = 3	1.64 ± 0.559	2.24 ± 0.671	0.592	< 0.001

6.	Do you think all residents should do at least one QI project during the 4-year residency training? No = 1 Unsure = 2 Yes = 3	2.83 ± 0.526	2.96 ± 0.255	0.132	0.011


**Table 4:** QI projects completed and presented by residents in training.

Project Title	Outcome

Time for change in the practice of in-patient oxygen therapy: A period limited, multidimensional quality improvement project	Poster presentation at IHI International Forum on Quality and Safety, Copenhagen, April 2020

Quality improvement project for improving in-patient glycaemic control in medical patients admitted with type 2 diabetes	Poster presentation at 55th Annual Meeting of the European Association for the Study of Diabetes Barcelona, Spain; 16–20 September 2019

Acute Kidney Injury: What we need to do in the first 24 h	Oral presentation at the 4th International General Internal Medicine Conference and American College of Physician Conference Oct. 2017 Doha, Qatar

Residents’ documentation of admission notes in the electronic medical record: “Are we compliant with hospital policy.”	Poster presentation at IHI Middle East Forum on Quality Improvement and Patient Safety – March 2018

Improving the standard of discharge summaries: Do we communicate properly?	Poster Presentation at IHI Middle East Forum on Quality Improvement and Patient Safety – March 2018


## References

[1] https://www.who.int/news-room/fact-sheets/detail/patient-safety.

[8] https://www.who.int/patientsafety/education/curriculum_guide_medical_schools_teaching_slides/en/.

[16] http://www.ihi.org/topics/improvementcapability/pages/education.aspx.

[22] https://www.facs.org/education/division-of-education/publications/rise/articles/six-steps.

